# Nursing students doing gender: Implications for higher education and the nursing profession

**DOI:** 10.1111/nin.12516

**Published:** 2022-08-11

**Authors:** Lesley Andrew, Ken Robinson, Julie Dare, Leesa Costello

**Affiliations:** ^1^ School of Nursing and Midwifery Edith Cowan University Joondalup Western Australia Australia; ^2^ School of Arts and Humanities Edith Cowan University Joondalup Western Australia Australia; ^3^ School of Medical and Health Sciences Edith Cowan University Joondalup Western Australia Australia

**Keywords:** autonomy, critical thinking, gender, higher education, maternal gatekeeping, nursing student, nursing workforce, social justice

## Abstract

The average age of women nursing students in Australia is rising. With this comes the likelihood that more now begin university with family responsibilities, and with their lives structured by the roles of mother and partner. Women with more traditionally gendered ideas of these roles, such as nurturing others and self‐sacrifice, are known to be attracted to nursing as a profession; once at university, however, these students can be vulnerable to gender role stress from the competing demands of study. A qualitative research design, guided by Gadamer's hermeneutic philosophy, explored the gendered behaviours and experiences of 22 women nursing students, all of whom had children and began university in a heterosexual intimate relationship. The findings reveal traditional ideas of gender were almost universal among participants, and these ideas had a significant influence on the nursing degree experience. Participants commonly prioritised family over the university and practiced maternal gatekeeping (prevention of male partner involvement in domestic work). These traditionally gendered behaviours, coupled with experiences of gender role stress, had a detrimental impact on participants' capacity to study and their personal wellbeing. The importance of these findings to the burgeoning nursing workforce shortage nursing is considered in terms of student retention and the supply of graduates into the profession. The implications to the nursing profession are also explored against the evidence that nursing students with traditional gender beliefs are less likely to develop as autonomous, critical thinking nurses compared to their gender‐egalitarian peers. The introduction of gender theory via critical pedagogy in the undergraduate nursing degree curriculum is recommended to enlighten and empower women nursing students and promote the competence, agility, and sustainability of the nursing profession.

## 1 BACKGROUND

The most recent Australian workforce planning projections have forecast a shortfall of 85,000 nurses by 2025 and 123,000 by 2030 (Health Workforce Australia, 2014). The implications of this workforce shortage to population health are dire, especially for communities and groups that experience disadvantage and marginalisation. People living in rural and remote Australian locations already face the consequences of significant nurse shortages, which have impacted accessible patient care. This situation is especially concerning for Australia's First Nations People, who already experience marked inequity in health outcomes (Australian Institute of Health and Welfare, 2018; Cosgrave et al., 2018). Further shortages therefore hold important implications for health equity and social justice in Australian health care.

A key strategy to mitigate this workforce shortfall is the attraction of new students into nursing and their support to successful graduation as competent, agile and critical thinking individuals. The first step to achieving these goals is to understand the changing nursing student demographic and their university experiences. Australian nursing students are increasingly mature age; more than half now begin their degree aged over 20 years (Department of Education and Training, 2018). Originally a profession for young single women, school leavers are turning to careers in other health professions that attract higher pay and prestige (Liaw et al., 2016; Price et al., 2013).

The continued increase in the proportion of mature‐age women (aged 21 years or over at the start of the degree) who study nursing in Australia (Department of Education and Training, 2015) suggests more nursing students than ever before have family commitments of their own (partners and children). The most recent Australian Bureau of Statistics (ABS) data on university student living arrangements reveal an average of 30% of these students (of all ages) live with a partner (ABS, 2013). This article explores the complex and contradictory gender dynamics within these students' families, the nursing profession and higher education, and their combined influence on the university experiences and opportunities of this rising cohort of women nursing students.

The following discussion introduces the importance of gender on the nursing student's university experiences and professional development through the theoretical lenses of gender ideology, maternal gatekeeping and the gender dynamics within the nursing profession.

### 1.1 Sex, gender and gender ideology

The study considers how gender influences the university experiences of cis women. Despite commonly being referred to interchangeably, ‘sex’ and ‘gender’ are not the same. A person's biological sex is defined by their chromosomal, hormonal, reproductive and other anatomical factors (World Health Organization [WHO], n.d.). Although other sexes exist (such as intersex), most people are identified as biologically male or female (Muehlenhard & Peterson, 2011). Gender is less definable and more of a continuum than sex and is a socially and culturally informed attribute that influences behaviours, roles and practices in relationships and in wider social interactions. As a social construct gender is in fact often regarded as something people ‘do’ rather than ‘are’ (West & Zimmerman, 1987). Gender is dynamic, as cultures and societal ideas change and shift, so do ideas of gender.

Despite the fluidity of gender, distinct attributes continue to be used to ascertain ‘male’ and ‘female’ gendered behaviours and to describe gender norms. The Male Role Norms Inventory, for example, devised by Levant (1992), defines masculine norms as: avoidance of femininity, fear and hatred of homosexuality, self‐reliance, aggression, achievement/status, non‐relational attitudes towards sexuality, and restrictive emotionality, whereas the Feminine Norms Inventory (Mahalik et al., 2005) identifies female gender role norms as: nice[ness] in relationships, thinness, modesty, domestic, care for children, romantic relationship [investment in] sexual fidelity and invest[ment] in appearance. Despite advancement in many areas of opportunity for women, the global persistence of gender stereotypes can be demonstrated through contemporary research. A study of 629 USA participants by Hentschel et al. (2019), for example, reported male participants saw themselves as more antigenic than women, and female and male participants described women as less assertive and weaker leaders than men.

It is generally well understood that internalised ideas of ‘being male’ and ‘being female’ (in terms of social norms and expectations) influence behaviours and interactions within the home. Individuals with traditional gender ideologies tend to follow traditional male/female roles in the family setting (Davis & Greenstein, 2009). In these relationships, the woman carries the domestic burden, the man and the financial responsibility. In contrast, individuals who align with egalitarian gender role ideology have a more flexible, fluid approach to family roles and are less influenced by traditional ideas of gender (Kulik & Tsoref, 2010). Within Australia, the former situation appears to dominate, with women spending around twice as much time on housework and childcare activities than their male partners (ABS, 2021).

These ideas of gender have global implications for the opportunities of women and men. Gender is a hierarchical social construct; in situations where traditional ideas of gender dominate, women experience subjugation and disadvantage across a wide range of life opportunities including education achievement, career opportunities and health outcomes (WHO, nd).

### 1.2 The nursing student, traditional ideas of gender and gender role stress

A somewhat dichotomous and contradictory situation of ‘gender‐based expectations and identities’ awaits the nursing student as they embark on their undergraduate degree towards their nursing career. The origin of the profession itself is based on traditional gender ideology with the female seen as a self‐sacrificing individual in ‘her natural state’—the caring role (Kellett et al., 2004). To some extent, the profession continues to function in an environment that reinforces this traditional gender ideology, with the nurse expected to serve under a medical system, noted for its institutionalised misogyny (Becker et al., 2022; Lewis, 2022). Nursing research reports that women students are often attracted to the profession by its ‘traditionally female’ characteristics of caring and nurturing (Price et al., 2013, p. 310) and tend to hold traditional ideas of gender themselves, with family prioritised over personal needs (Andrew et al., 2020; Burton, 2020; Kellet et al., 2004).

Gender role stress, described as the stress experienced by an individual when placed in a situation that limits their ability to follow behaviours dictated by their personally held gender beliefs, can impact a nursing student's capacity to function and achieve in their degree (Kargin et al., 2021; McLaughlin et al., 2010). As nursing students, these women must navigate a higher education system and degree curriculum that expects them to behave in a ‘traditionally male’ way, with the capacity to prioritise study over family (Andrew et al., 2020; Currie et al., 2000). While a caring and nurturing approach remains central to quality nursing practice (Rolfe, 2015), there is also an increasing requirement for the nursing student to develop ‘traditionally male’ attributes of leadership, critical thinking capabilities and autonomous practice, and the capacity to prioritise career and personal success. Such situations may place considerable stress on the woman student with beliefs and family routines that align with the traditional idea of ‘being female’.

Critical thinking attributes also support the nurse's capacity to work effectively and sensitively across different social groups to deliver equitable care (Einhellig et al., 2016). Traditional gender role ideology can act as a barrier to the student's development of these essential nursing qualities. There is also evidence that students with traditional gender ideology hold a less developed perception of nursing professionalism (Park et al., 2019).

### 1.3 Maternal gatekeeping

The theory of maternal gatekeeping provides a useful way to understand gender role stress in nursing students who are also mothers and partners in a heterosexual intimate relationship. The theory is based on the frameworks of the social construction of gender and gender ideology and their influence on a woman's beliefs and actions, rather than the influence of the woman's biological sex. The theory, introduced by Allen and Hawkins (1999) and validated by Gaunt (2008), conceptualises women's gendered behaviours of restricting fathers' involvement in childrearing (gate closing). Allen and Hawkins divide this behaviour into three dimensions. ‘Differentiated family roles’ describes the belief that gender roles are necessarily separate within the heterosexual intimate relationship. ‘Standards and responsibility’ describe the imposition by mothers of rigid expectations and standards around childcare and housework and the criticism of their partners' attempt to share in this work. ‘Maternal identity confirmation’ describes the women's sense of validation from achieving and being seen to achieve the idea of the good mother, who sacrifices her own needs for those of her children. Allen and Hawkins (1999) argue that this behaviour offers the woman a feeling of self‐worth within an otherwise patriarchal society.

In 2013, Puhlman and Pasley revisited maternal gatekeeping theory from a feminist perspective, informed by family systems theory, broadening it to consider the wider structural issues that pressurise women to undertake these behaviours. They also introduced the idea of a dynamic gatekeeping model, which includes gate opening practices, where mothers begin to encourage and support male partners' domestic responsibility.

The idea that gender as a social construct, rather than innate biological factors, influence gatekeeping practices has been reinforced by a study of gatekeeping behaviours among heterosexual couples and same‐sex couples (women and men) during their transition as adoptive parents (Sweeney et al., 2017). As expected, women in a heterosexual relationship displayed more gatekeeping practices than the other family patterns, with men in these heterosexual relationships displaying minimal gatekeeping behaviour. The behaviour was however also observed in same‐sex male couples, and less so in same‐sex women couples, indicating the lack of a biological link to this practice and supporting the idea of the association between gendered relationship dynamics and power imbalances. These researchers postulated the higher rate of men than women in same‐sex relationships who practiced gatekeeping related to the lower levels of egalitarian domestic role division in these male relationships, as well as the pressure to exaggerate this behaviour from societal prejudice that positions women, not men, as ‘natural’ or ‘competent’ parents.

The profound and pervasive influence of gender and gendered expectations within society may have important implications for the increasing proportion of older women who now study nursing in Australia, as well as the sustainability of the nursing workforce that is equipped to understand and meet diverse population needs. Despite this, the impact of the gendered behaviour of maternal gatekeeping on the nursing student experience, a highly gender‐traditional group (Andrew et al., 2020; Burton, 2020; Kellet et al., 2004), has not been considered in the literature. This article explores this practice and its influence on student achievement and wellbeing. The potential consequences of gendered behaviors for the nursing profession and workforce are also discussed against the rising requirement for a highly skilled and autonomous profession and the ongoing nursing workforce shortage (Health Workforce Australia, 2014).

The purpose of this study is therefore to explore and develop an understanding of the experiences of women nursing students and the complex and sometimes contradictory influences of gender expectations on their university experience and ability to progress and achieve in their undergraduate degree.

## 2 METHODS

### 2.1 Design

The study was qualitative, guided by Gadamer's hermeneutic philosophy (Gadamer, 1989), which holds that the path to ‘truth’ in the social sciences occurs through an open relationship between experience, participant and researcher conversation, and theory, rather than the testing of prior knowledge. Gadamer's hermeneutic philosophy sits within the social constructivist research paradigm. For Gadamer, truth is fluid and changing, and is created through an understanding of the rich and complex nature and experience of ‘being’ for humans, across multiple human perspectives (McManus Holroyd, 2007).

The study explored the meaning of women's experiences at university, from their own perspectives. Participants were invited for interviews in their second year of study and again in their final year. In taking a two‐phase approach to data collection, the complex and changing nature of maternal gatekeeping behaviour across the degree was captured.

### 2.2 Setting, recruitment and participants

In‐depth semistructured interviews were held in 2015 and 2016 across two campuses of an Australian university in the School of Nursing. The study recruited a purposive sample of cisgender women nursing students who identified as female, and who had begun university in an intimate heterosexual relationship. Women who did not live with a male partner were excluded. This paper reports on the 22 women in the sample who had children who lived with them during their degree.

The study was promoted via hardcopy posters in the School of Nursing clinical areas and the university's online learning platform student community site. Interested students were sent a participant information letter and consent form to support informed decision‐making. All interviews took place in person (face‐to‐face) with the principal researcher as an interviewer, at a time and place convenient to the participant. All but three interviews were conducted on the university's campus site, the remainder took place in a café or park near the participant's home. On‐campus interviews were conducted away from the School of Nursing building to ensure participant privacy and encourage openness. Only interviews with students who completed their degree were included in the data analysis so that the influences on their experience were captured across the degree journey.

The study was approved by the University Ethics Board before participant recruitment. As the nature of conversations may have led to the recall of difficult experiences, such as stressful situations within the university and family, participants were reminded at the start of each interview that they did not have to discuss anything that made them feel uncomfortable. The contact details of the university's free student counselling services were also shared.

### 2.3 Interviewer reflexivity

In alignment with Gadamerian hermeneutic philosophy (Gadamer, 1989), the researcher's own experiences are an integral part of the creation of meaning in this study; omitting this practice may lead to unchallenged and ongoing reliance on the researcher's preconceived ideas during data collection and analysis, which in turn can ‘deprive the text of the opportunity to manifest its truth’ (Geanellos, 1998, p. 156) and reduce credibility. Research findings were developed through consensus and reflection rather than the researcher's personal view, bias or a mere description of the participant's words. The data collection and analysis process was supported by the interviewer's use of a reflective diary before and after each interview, in which they recorded and reflected on their own opinions and prejudices and how these were challenged with successive interviews. This process guided the researcher's progression from a position of prejudice to a state of consensus of understanding and the creation of meaning. It is important to note that the participants' perspectives were sometimes challenged and ran contrary to the researcher's initial assumptions. By reflecting on these different views, and revisiting theoretical works, the researcher develops a meaningful understanding of the participants' situations, perspectives and social reality.

Two of the researcher's initial prejudices challenged by this process were the assumption that participants would wish and expect to relinquish some of their domestic responsibilities when starting their degree; for many of the participants, this was not the case. The second centred on the researcher's idea of how a ‘supportive’ partner would behave, which sometimes differed from the participants' ideas. The researcher was able to reframe their understanding by actively listening to participants' experiences and reframing them through the lens of maternal gatekeeping and traditional gender ideology.

### 2.4 Data collection and analysis

Gadamer sought to create understanding and meaning from conversation through the interplay of ideas and voices of the researcher and participant and the development of consensus (Gadamer, 1989). This can only be done if the researcher maintains an open mind and encourages participants to share their own perspectives and beliefs however dissimilar they are to their own (Palmer, 2001, p. 11). To support openness, a nonjudgemental, nonhierarchical and inclusive interview approach was employed, in which the interviewer shared their own experiences as appropriate. While concurring with Gadamerian philosophy, this approach is also particularly important in studies where the woman's perspective is sought. This, according to feminist scholars, can only be achieved through the development of a nonhierarchical conversation, cooperation and coconstruction of meaning between the woman and the researcher (Denzin & Lincoln, 2013; Oakley, 2000).

Participants were invited to interview in the second and final years of their degree journey. Eighteen of the 22 completed both interviews, with four unavailable for their second interview because of clinical practice commitments. The lead author, who was not employed in the School of Nursing and therefore not involved in any form of power relationship with the participants, conducted the interviews, which were audio recorded with participant consent. Conversations explored participants' university experiences and the factors that influenced these experiences. All interviews were conducted and transcribed by the interviewer, to ensure continuity of understanding and ongoing immersion in the text. A transcribed interview summary was returned to each participant for the purpose of member checking with minor alternations made to two interview transcripts. Data collection and analysis took place concurrently, with findings informing successive conversations.

Participant recruitment continued until the analysis of four consecutive interviews led to no new development of codes or expansion of themes. Around 40 h of transcribed data were organised using NVivo software. Inductive data analysis took place through open coding and theme development. Prolonged engagement during data analysis was enhanced through the use of Gadamer's concept of the hermeneutic circle (Gadamer, 1989) where an iterative back and forth ‘play’ took place between the participant conversations, the researcher's prejudices and relevant theory.

## 3 FINDINGS

### 3.1 Demographics

All 22 participants were undergraduates in the Bachelor of Science (Nursing). Their ages at the time they began their degree ranged from 23 to 48 years (mean 39 years). All began their degree while in an intimate heterosexual relationship. The average length of these relationships at the start of the degree was 12 years. All participants were mothers of children aged under 18 years who were living at home.

The themes developed were organised against the three categories of maternal gatekeeping described by Allen and Hawkins (1999), and against a further category ‘opening the gate’ introduced by Puhlman and Pasley (2013). The findings demonstrate the influence of traditional ideas of gender on participants and their partner's behaviours and expectations and their impact on the woman's family life and their academic achievement and wellbeing, and the influence of education on these traditional ideas. Direct quotes from interviews are shown as (pseudonym, age at the start of the degree, no. of children).

### 3.2 Differentiated family roles

It became evident from conversations with the women in this study that their family lifestyles and expectations fitted within traditional ideas of gender. Participants described distinct patterns of behaviour in the intimate relationship that followed traditional ideas of the woman and man's role within the family and wider society. Chantelle (38, ch. 3) explained, ‘My husband was very much the breadwinner, bringing in the money, and I was the housewife’. Just two of these women (Ruth, 38, ch. 1 and Brenda, 46, ch. 2) described a more egalitarian sharing of roles within the intimate relationship, where domestic work was divided between couples according to time available rather than to traditional gender roles. In the vast majority of relationships, the women took on sole responsibility for childcare and housework. Lauren (32, ch. 2), for example, recalled ‘He would never do the dishes, he doesn't even know what a vacuum cleaner was’.

Participants explained how they went to great efforts to maintain these divisions once they began their nursing degree. Many described their attempts to protect their partners and children from the intrusion of the university on family workload dynamics and to maintain the prioritisation of their family's needs over their own academic and carer ambition. This perspective is illustrated by Jilly (45, ch. 2), who stated, ‘I don't want my study to take over everything if you see what I mean. I don't want them to feel that uni is more important than them’. Efforts to shield the family from the intrusion of the university meant the participants rarely asked their partners and children for help with domestic tasks; many recounted how they would forgo sleep and rest to ‘fit‐in’ study, in the belief that family must be prioritised at all times. Participants also described how they set high personal standards around housework and childcare. Some described an almost ‘intrinsic’ need for domestic perfection, including Rebecca (43, ch. 4) who stated, ‘It's me I think I must have a bit of OCD I can't sit down if I see things that need doing… Yes and I feel like I've got to do that first and then I'll sit down’.

The women's days were organised so that university study was attempted after domestic work was completed. Maureen (38, ch. 2) said, ‘I'll take my son to school, then I'll nip to the shop if I need to go to the shop and I'll put a wash or come home and peg the washing out. Once that's done then I'll go and sit and do my uni stuff’. While these women described an awareness that this standard setting and prioritisation was detrimental to their nursing degree progress, they also recalled how, at least in the early stages of the degree, this behaviour continued, resulting in a heavy competing burden of study and domestic work:

I think it has (had an impact on study) because I end up having to stay up 1 or 2 in the morning getting assignments done study stuff like that. I should just walk away from the housework but I can't do that (Jilly, 45, ch. 2).

Compounding this behaviour was the view that partners were unable or unwilling to meet these standards, and often criticised their partners' efforts to complete domestic work. Many recalled stories of their partner's failed attempts to manage household tasks, including Lauren (38, ch. 2) who said, ‘He wasn't a particularly good cleaner. I remember the first time he cleaned the toilet he scrubbed the inside before the top and I was like “Oh my god I don't want to sit on that toilet ever again”’. These women sometimes discussed how they had ‘banned’ their partners from domestic work because they were not able to complete it sufficiently well. Ros (32, ch. 2), for example, described her partner's practice of not putting enough washing in the machine meant he was, ‘Not allowed to do the washing’. He was also not trusted to take on childcare, with Ros explaining how he was unreliable when asked to pick up the children from school, saying, ‘He'd always forget, I can guarantee’.

These findings illustrate the ingrained gendered expectations and differentiated roles of both partners in the intimate relationship and how the gender role stress initiated by the competing expectations of the university influenced the women's capacity to study. As more women study nursing at later stages in life, this situation is likely to affect an increasing proportion of students.

### 3.3 Maternal identity confirmation

Participants described how their role as a mother influenced their prioritisation of family before their academic goals. This idea of motherhood was clearly informed by their expectation that a mother's needs came last; clearly aligning with traditional female gender norms, illustrated by the following conversation with Sherry (47, ch. 2):

Sherry: I think I still have that mind‐set that I have to hold it all together and what I want comes last.

Interviewer: Right do you think that's a female thing?

Sherry: I think it's a mother thing.

Interviewer: So, you've got set roles and you need to make sure they are all fulfilled even though you are taking on another role?

Sherry: Yes you've just got to fit that in… If I had all my time just to study no doubt I could do better … far better but …normal life doesn't allow you to do that.

Over time, efforts to shield their family became more difficult as the expectations of the university competed for the participants' resources. This situation created a situation of role conflict for these women which led to feelings of guilt and distress; this ultimately impacted their capacity to engage in the range of study opportunities offered by the university. Maureen (38, ch. 2), for example, stated, ‘Sometimes I feel guilty about being at uni at the weekend and evenings, so I don't come in much. I feel I should be at home’.

Participants explained how their partners tended to hold similar views about the woman's primary role as a child carer and domestic worker. These partners were reluctant to share this ‘traditionally female’ role. Georgia (23, ch. 1) described her partner's perspective with the words, ‘In his head that's my job… that's part of my role as the female in the home’. Partners' expressions of unhappiness at the intrusion of the university into the family routine compounded the women's guilt and affected their overall wellbeing:

That first year honestly was horrendous. I can remember crying just because the pressure of uni, then he'd make me feel bad. I'll always remember him saying something like “it's coming before the kids”. Then I'd think “oh my god, I'm supposed to be a mum”. I felt horrible (Candice, 40, ch. 3).

Although this research focused on students who completed their degree, many participants recalled how the competing stresses of the university and the family have caused them to move to part‐time study, online study and in a few cases to defer or intermit in their course. Many recounted peers who had given up their degree. Rebecca (43, ch. 4), for example, described how her friend, also a mature‐age woman student, had found the juggling of family and university impossible, saying ‘if it's going to be like this for three years… I can't do it’.

These student experiences demonstrate the impact of conflicting demands on the woman student and the way they can jeopardise the student's access study opportunities, and with this their opportunity to progress and achieve.

### 3.4 Gate opening practices

At the start of the degree, the women's identity as a mother had been paramount. As Anne (38, ch. 3) explained, ‘Before I started this … my life revolved around the kids and the school’. While many of the women in this study strove to maintain their efforts to ‘keep the gate shut’ throughout the degree, others began to question this behaviour and the reasons behind it. Catalysts for this seem to include the development of a competing identity. Over time, some described how they began to enjoy new identities as a student and as a future nursing professional, which brought them a sense of wellbeing, including Brenda (46, ch. 2) who stated, ‘I really enjoyed it; I got this new lease of life. All these new people and they see you as someone completely different, and I just really loved it’.

Most of the women described how they had been employed in casual, part‐time and auxiliary jobs before their degree to fit around the family. Participants commonly recounted how their developing vision of themselves as a nurse and the promise of a career as a health professional had supported their ability to manage the stress and guilt they experienced as a result of the conflicting demands of motherhood and student life. Sharon (32, ch. 2) said, ‘I think the biggest thing that's kept me here is knowing that this (becoming a nurse) is what you want’. The increasing importance of these new identities helped these women to shift their priorities and make more room for the university in their lives. Some women began to overcome feelings of guilt to share the domestic load with their partners:

As time passed, I've probably been able to put myself first instead of everyone else. I probably am not as hard on myself. Now when he's (husband) been having a whinge that I haven't cooked tea, whereas before I would feel guilty, now I think “too bad” (Maureen, 38, ch. 2).

Children were also expected to take more responsibility in the home:

I've got baskets with the kids' names on them. So, if I pick something up I just chuck it in the basket. That's their job to tidy it up now. Whereas I would have done that before. Towards the end of my degree, I've just said “do you know what, you've got to fit around me” (Ros, 32, ch. 2).

An unhelpful consequence of this change in participants' behaviours and expectations came from partner's responses to these changes in role dynamics. Some women described how the relationship tension this created led them to avoid asking for help wherever possible. Rebecca (43, ch. 4) described her partner's expressions of unhappiness prompted her to forgo rest and sleep to complete all the family domestic work, ‘He has a moan about that so it's just not worth it. So I'll just wait until the evenings and do it then when the kids are asleep’. For some participants, the resulting relationship tension escalated into relationship conflict during the degree (see Andrew et al., 2020). For others, however, opening communications around expectations and needs facilitated the process. Frankie (32, ch. 2), for example, explained, ‘If you ask them then they will, most of the time. Even if you say, “can you do it now so I'm not stressing about it”. You've just got to be open’.

Participants described how explicit communication of expectations was needed with partners who ‘just didn't see’ the load they had been carrying and its effects on their wellbeing and academic work. Articulation of needs within a relationship, however, depends on the woman recognising these needs and her rights to pursue them. Nurse education can play a part in this enlightenment within the curriculum.

## 4 DISCUSSION

These findings are now interpreted through the lens of traditional gender ideology and gender role stress and through the theory of maternal gatekeeping described by Allen and Hawkins (1999) and by Puhlman and Pasley (2013). The influences on gate‐closing practices and later gate opening behaviours are considered and the consequences of both behaviours for the women are discussed. The wider implications of traditional gender ideology for the nursing profession and workforce are explored. Recommendations to nurse education are drawn from these discussions.

### 4.1 Participants' traditional ideas of gender and maternal gatekeeping behaviours

The participants' behaviour reflected all three dimensions of maternal gatekeeping described by Allen and Hawkins (1999). It appears they practiced as ‘traditional gate blockers’ at the time they began the degree, which is high control, low encouragement and high discouragement of male partner involvement in childcare and housework (Puhlman & Pasley, 2013).

Differentiated family roles and traditional ideas of gender were evident in most of the intimate relationships. Life revolved around highly traditional ideas of women and men, a finding that concurs with previous nursing student research (Andrew et al., 2020; Price et al., 2013). ‘The setting of high and inflexible standards and expectations around housekeeping and childcare’ (Allen & Hawkins, 1999) was evident. Criticism of and dissatisfaction with male partners' efforts limited partner support, reflecting findings in previous gatekeeping research with married mothers (Kulik & Tsoref, 2010).

The ‘validation of maternal identity’ dimension, in which traditional ideas of the female and mother provide a source of identity, satisfaction and self‐esteem (Allen & Hawkins, 1999; Gaunt, 2008) was also important to the participants. Hauser's argument (2012) that sharing of these duties could potentially erode ‘sense of indispensability’ brought about by this identity among women with traditional gender ideology, and in doing so, diminish the self‐esteem derived from it, appears relevant to this student group, most of whom had worked in auxiliary roles with limited power and opportunity.

The experiences of gender role stress described by participants as the university began to compete with their family for their time have been noted previously among nursing students in the scholarly literature, where these experiences were found to contribute to low self‐esteem and high rates of attrition (Kargin et al., 2021; McLaughlin et al., 2010). The present study is the first to interpret this stress through the lens of maternal gatekeeping. In doing so, it provides a deeper understanding of the gendered drivers behind the women student's behaviours and experiences of guilt and distress, and inability to fully meet the demands of university study. While the study focused on students who continued to graduation, many of the participants described how this stress had disrupted their progression and recalled peers who had become overwhelmed and had discontinued. These findings provide a worrying insight into the lives of women nursing students with family commitments at a time such students increasingly make up the supply of the nursing workforce.

### 4.2 The family and the nursing profession as reinforcers of traditional gender roles and catalysts of gender role stress

In their seminal work, Allen and Hawkins (1999) reported just 22% of their participants adopted gatekeeping (gate‐closing) behaviour. Although not designed to seek statistical findings, the present study found a clear majority (20 of the 22 participants) adopted this behaviour, at least in the early stages of their degree. Like the women in Allen and Hawkins study, all the participants were in a heterosexual intimate relationship and had children. Described by Davis and Greenstein as ‘highly gendered institution[s]’ (2009, p. 95), ideas of gender between couples tend to become polarised within such relationships, with women and men assuming distinctly ‘female’ and ‘male’ roles, a situation that can become further polarised if the couple have children (Gaunt, 2008). The finding that partners reinforced gate‐closing behaviour through their reluctance to undertake domestic tasks adds credence to the argument by Puhlman and Pasley (2013) of the complex structural factors that play a part in maternal gatekeeping, including the expectations of a patriarchal society and the pressure it places on men and women to conform to traditional gender norms. This finding upholds criticisms of victim blaming (Puhlman & Pasley, 2013) aimed at scholars who place sole responsibility for maternal gatekeeping behaviours with the woman, and ignores power relationships between the genders, and wider structural forces.

The greater prevalence of gate‐closing behaviours in the present study may also be due to the traditionally gendered nature of nursing, which subscribes to the traditional ideas of the female and attracts students who align with such views (Andrew et al., 2020; Price et al., 2013). Perhaps more than any other profession, nurses nurture and provide care for others, while submitting to a dominant patriarchal medical profession and to structured hierarchies within their own profession (Burton, 2020; Kellet et al., 2004; Lewis, 2022).

This proliferation of the image of the nurse as a martyr not only attracts women students who are somewhat destined to experience gender role stress at university but also deters other women students who are discouraged by such traditional ideas, and who in theory are therefore more able to manage the demands of the degree. Research by Price et al. (2013) and Liaw et al. (2016) demonstrates that school leavers in particular reject the idea of nursing as a future profession because of the perceived subservience of the nurse. Nurse education has an important role to play in attracting more students of all ages to the profession. It appears that one way of doing this is through the alternation of the perception and reality of nursing from the ‘angel’ to the critical thinking and autonomous professional with the capacity to make a change.

### 4.3 Higher education, competing identities and gate‐opening behaviours

Although not universal, some participants described gate‐opening behaviours during their degree. Puhlman and Pasley (2013) reported catalysts for this change in practice to include divorce and evolving issues within the family such as domestic abuse and illness. In the present study, this behaviour seemed to be prompted, at least in part, by the development of new identities and changing priorities. Over time, the women's nursing and student identities emerged and competed for space with their identity as a mother. For these women, these identities provided a new source of self‐worth, reducing the intensity of the importance of their maternal identity described by Allen and Hawkins (1999), Gaunt (2008) and Hauser (2012).

Gender ideology, which underlies gatekeeping practices, is a dynamic concept, influenced by an individual's interactions across disparate social contexts (West & Zimmerman, 1987). Nursing students encounter a myriad of social and cultural situations during a degree in which they are introduced to the concepts of social justice and equity (WHO, 2020). Higher education itself is known to be a transformative experience that supports the development of more egalitarian ideas of gender (Baxter, 2014). This transformation was demonstrated among the participants who began to question their previously held traditional gender ideology.

Paradoxically then, while the traditional image of nursing attracted these participants and reinforced the traditional idea of women and gate‐closing behaviours, for some participants, the degree itself, and the wider higher education experience served to promote their questioning of these norms and the shift to gate opening practices. A further paradox for some participants was the outcome of this behaviour change, which lowered their feelings of guilt and made space for their degree progression but led to relationship tension as partners resisted requests to share the domestic load (see Andrew et al., 2020).

Nurse education has the potential to support women students who are placed in this paradoxical situation to recognise and value their own needs and to set expectations within the family by communicating these needs in the early stages of the degree.

### 4.4 Issues of gender and the wider implications for the nursing profession

Gender role stress, evident among participants and described more widely in nursing research, is associated with nursing student attrition (Kargin et al., 2021). As more nursing students begin their nursing degree as mature‐age women, the impact of gender ideology and gendered expectations on student experience and their capacity to progress and succeed at university requires attention within nurse education. Women nursing students need support and guidance to recognise the impact of their gate closing behaviour (Gaunt, 2008) on their capacity to succeed as well as their personal wellbeing. The ongoing threat of a nursing workforce shortage heightens this requirement (Health Workforce Australia, 2014). Efforts within the nursing curriculum to raise student awareness of the barriers traditional gender roles and associated behaviours represent to their progression and achievement is central to student retention and workforce sustainability.

Research evidence suggests these efforts are also needed to ensure an assertive and agile nursing identity. Traditional gender ideology has been identified as a barrier to the nursing student's development of autonomy, innovation, initiative and leadership, all prerequisites to safe high‐quality patient care (Burton, 2020; Johnson et al., 2012; Park et al., 2019) and has led to calls for an awareness raising in nursing student education of influence of traditional gender norms on the autonomy and competence of the nursing student and future professional practitioner (Ayten, 2019; Burton, 2020; Kargin et al., 2021; Park et al., 2019).

Agile and accessible nursing practice in an ever‐changing and multicultural environment such as Australia also requires an understanding of social justice and its impact on health outcomes. For this reason, nursing academics have called for a stronger emphasis on the teaching of social justice within the nursing curriculum (Boutain, 2008; Einhellig et al., 2016), the recognition of gender as a key influence on the health and safety of women (Basar, 2017) and the nurse's pivotal responsibility in the achievement of equitable health outcomes for women (United Nations, 2015). To truly understand the importance of social justice, however, the nursing student requires an awareness of the significance of gender within their own lives and their opportunity to succeed as a student and future nurse. The recommendations below outline ways this can be achieved in practice.

## 5 LIMITATIONS

As a qualitative study, the generalisability of these findings to a national or international stage is limited by small participant numbers. However, the social construct of gender and its influence on women's opportunities supports the relevance of these findings to women in nursing and higher education worldwide. This study is limited to the experiences of cisgender women who are in a heterosexual relationship. The authors acknowledge the importance of future research into gender norms and the experiences of cis men and LGBTQ + student experiences, which have been neglected to date.

## 6 RECOMMENDATIONS

Nurse education has changed in recent decades from a model where ‘the student is a passive recipient of information, to a critical model where the student is engaged in the process of developing autonomy and empowerment’ (Allen, 2010, p. 33). This study adds to calls from scholars and researchers to embed gender theory within this critical model so that nurses can be enlightened and enact change (Ayten, 2019; Burton, 2020; Kargin et al., 2021; Park et al., 2019). Freire (1973) has been recommended as a guide to support this practice by nursing scholars Roberts (1983) and by Allen (2010), because of its capacity to help students from subjugated or oppressed groups to question and challenge unhelpful regimes. In acquiring critical consciousness, future nurses can enact change to support degree progression, enrich the profession and protect the future workforce supply.

A range of studies have reported how Freire's (1973) ideas of critical pedagogy can be incorporated into student education. Duffy and Powers (2018), for example, describe the use of reflective practice and the ‘Theatre of the Oppressed’ (use of theatre to engage students in real‐world situations of oppression), to expand the student's understanding of social justice and their role in its promotion. The use of the Theatre of the Oppressed approach has also been used to create a classroom environment in social work teaching where students can ‘engage in the intellectual and emotional growth needed to grasp social justice concepts and practical implications’ (Garcia et al., 2019, p. 669). This personal growth relies on the sharing of personal histories and relationships to and experiences of dominance, marginalisation and empowerment (Jeyaraj, 2020). More recently, Van Bewer et al. (2021) found the inclusion of the Theatre of the Oppressed method in undergraduate nursing studies supported the student's personal growth and appreciation of social justice as an integral aspect of their practice.

Although outside the scope of this study, it is important to note the link between traditional gender ideology and bullying in the nursing profession, with women nurses with less developed professional identities and assertiveness skills reported to rely more on passive‐aggressive behaviours to gain some sort of control and autonomy in their work (Akella & Seay, 2022). Future research on bullying in the nursing profession may benefit from an understanding of this relationship.

It is also important to conclude with an acknowledgement that wider structural factors deny nurses their autonomy, such as the portrayal of the nurse as a submissive and self‐sacrificing martyr by media and political leaders and the persistence of misogyny in the health system and wider society (Becker et al., 2022). While an empowered nursing workforce can go some way to achieve this, a change in the nursing degree delivery itself is required, to one that provides flexible and accessible learning opportunities to all (see Andrew et al., 2022). Wider societal and political changes are also needed to facilitate the required changes recommended here.

## CONFLICT OF INTEREST

The authors declare no conflict of interest.

## Data Availability

Data are available on request due to privacy/ethical restrictions.
